# Patient-specific estimation of distal lung morphometry using aerosol deposition data

**DOI:** 10.3389/fphys.2025.1611545

**Published:** 2025-07-29

**Authors:** S. G. Karthiga Devi, Alladi Mohan, Kalawat T.C, Mahesh V. Panchagnula

**Affiliations:** ^1^ Department of Applied Mechanics and Biomedical Engineering, Indian Institute of Technology Madras, Chennai, India; ^2^ Department of Environmental Health Engineering, Sri Ramachandra Institute of Higher Education and Research, Chennai, India; ^3^ Department of Medicine, Sri Venkateswara Institute of Medical Sciences, Tirupati, India; ^4^ Department of Nuclear Medicine, Sri Venkateswara Institute of Medical Sciences, Tirupati, India

**Keywords:** personalized medicine, gamma scintigraphy, regional deposition, mucocilliary clearance, inter-subject variability

## Abstract

**Background:**

Current imaging and diagnostic methods are unable to visualize distal lung beyond the seventh branching generation, despite its critical role in lung function. Inter-individual variability in lung structure influences aerosol deposition within the alveolar region. Understanding an individual’s lung dimensions enables the development of customized treatments. This constitutes the key motivation behind the study.

**Methods:**

This study reports a method to estimate morphometric parameters associated with the distal lung using aerosol deposition characteristics. Aerosol deposition characteristics are measured in a cohort of healthy human subjects using gamma scintigraphy. From this data, differences in the aerosol deposition from subject to subject are quantified. Using a mathematical model of aerosol transport, we demonstrate a novel procedure to estimate patient-specific lung morphometric parameters of the distal generations of the human lung and average alveolar diameter.

**Results:**

Morphometric parameters of the healthy subjects recruited in the study are determined. The lung volumes predicted using the morphometric parameters are unique and different for each subject as expected. The study also predicts the airway diameters and length at every generation for all of the subjects. From the predicted parameters for each of the individual, it can be clearly seen that the lung dimensions vary from individual to individual.

**Conclusion:**

The study demonstrates that measurements of aerosol deposition, especially in the distal generations, are a highly sensitive marker for inter-subject variability in lung morphology. The method presented in this study could be packaged into a walk-in lab test, the results from which could help physicians to personalize treatment and pulmonary drug dosage.

## 1 Introduction

The lung is one of the vital internal organs, which is continuously exposed to the external environment. This underlies the cause of several respiratory infections. According to the Global Burden of Disease (GBD) study, chronic obstructive pulmonary disease (COPD) and lower respiratory infections rank as the 3rd and 4th leading causes of global deaths, respectively (Naghavi et al., 2024). The projected deaths in 2030 due to these diseases are expected to remain almost in the same positions (Mathers and Loncar, 2006). Some individuals are more susceptible to infections, while others are more resistant. This variation in infection susceptibility in the lung can be attributed to several interrelated immunological, biological, environmental, genetic and lifestyle factors (Mizgerd, 2018). A relevant factor considered in this work is the variability in aerosol deposition patterns within the alveolar region.

Diagnostic tests such as spirometry cannot detect early-stage lung diseases as it primarily measures airflow, missing subtle lung abnormalities (Sylvester et al., 2021). Newer diagnostic methods using advanced imaging techniques, biomarker analysis, and breath analysis show promise, however; are not yet widely adopted in clinical practice (National Academies of Sciences E, Division H and M, Services B on HC, Techniques C on IN or ID or E, 2023; Naseem et al., 2024; Juneau et al., 2024). Most of the people with COPD or bronchial asthma remain undiagnosed and their respiratory symptoms remain untreated (Aaron et al., 2024). Moreover, recent research in respiratory disease treatment emphasizes the development of patient-specific prototype models using advanced imaging tools (Hong et al., 2023). However, creating and analyzing these models is highly complex, making it challenging to generate personalized models for every individual. In addition, there are no direct imaging modalities that are capable of providing dimensions of the small airways, which are lesser than 2 mm (Stenberg et al., 2025).

Few indirect metrics have been developed in late 1990s to measure the microstructure of the airways. Aerosol derived lung morphometry (ADAM) infers airway dimensions by measuring the gravitational settling of inhaled particles and estimates the effective air space dimension (EAD) as a function of volumetric lung depth (VLD; Brand et al., 1995; Zeman and Bennett, 2006; Zeman and Bennett, 1995; Brand et al., 1999). However, ADAM is not used clinically due to its requirement for repeatable and low breathing flow rates, which can be challenging for patients, along with uncertainties in particle penetration into diseased lungs (Löndahl et al., 2017). Airspace dimension assessment (AiDA) technique has been employed in the past decade to measure the lung dimensions. While ADAM is based on the particles that deposit by sedimentation due to gravity, AiDA is based on the recovery rate of inhaled nanoparticles (Petersson-Sjögren et al., 2021) primarily deposited by Brownian diffusion. The fraction of nanoparticles that deposit in the distal airspaces during the breath-hold is directly related to the size of lung airways. Studies have shown that airspace dimensions obtained using AiDA correlates well with diffusive metrics obtained from hyperpolarized 129Xe diffusion-weighted imaging of lung (Petersson-Sjögren et al., 2021), lung tissue density measured by magnetic resonance imaging (MRI; Aaltonen et al., 2018) and is also used in detecting early-stage emphysema (Aaltonen et al., 2021). However, neither of these methods have achieved the requisite level of validation and standardization necessary for clinical adoption in measuring lung morphometry.

Furthermore, the expense of sophisticated instrumentation necessary for quantifying nanoparticle recovery rates adds to the overall cost. For instance, monodisperse aerosol generators, differential mobility analyzers and condensation particle counters (CPC) are typically required to measure the AiDA (Jakobsson et al., 2016). These complex instruments are not generally available in a typical hospital setting accessible to the physician nor the patient. Radiation exposure is another challenge associated with advanced high-resolution CT (HRCT) or MRI imaging. While the radiation dose received by an individual may be below the thresholds set by regulatory bodies, it remains significant. In many cases, the dose is comparable to or exceeds the cumulative background radiation exposure over a period of 6 months (Radiology RS of NA and AC of. Radiation Dose). Combinations of imaging and processing are required to develop methods for measuring lung morphology. An optimal approach would therefore involve minimal radiation exposure, utilizing readily accessible imaging and aerosol generation equipment, and requiring relatively low computational resources for predicting lung morphometry.

Lung deposition of a drug can serve as an indicator of its local bioavailability, thus serving as a surrogate to identify the clinical response of inhaled drug (Newman, 2000). On the issue of drug dosage and bioavailability at the functional sites of the lung, inter-subject variability in drug deposition is a critical challenge that needs to be understood and overcome. Significant differences occur in particle deposition in different human subjects breathing the same aerosol, contributing to substantial inter-subject variability (Chan and Lippmann, 1980; Smaldone et al., 1989; Heyder et al., 1988). However, no data are available in the literature as to the extent of such variability. Importantly, alveolar deposition is sensitive to inter-subject morphometric variations of the lung (Karthiga et al., 2016), and the link needs to be understood. Specifically, the scientific community has not explored the contribution of inter-subject morphometric variation of the distal generations to variability in drug uptake. Effective aerosol treatment pathways needs to integrate both population-based models and patient specific strategies. Population based models provide the necessary dosing guidelines on the whole, whereas patient-based models are significant in integrating inter-subject variability in achieving the necessary dosing for the patient. Recent review on inter-subject variability (Kassinos and Sznitman, 2025) has highlighted the importance of this dual approach to maximize treatment outcomes with practical considerations.

Recent study (Christou et al., 2021) highlight the importance of anatomical variability in the upper respiratory tract and its impact on aerosol deposition. While it focuses on proximal airways using CT imaging, our study specifically attempts to address inter-subject variability in the distal lung geometry, extending into regions that are beyond the spatial resolution or accessibility of conventional imaging techniques. By leveraging aerosol deposition data and mathematical modelling, our approach provides a novel, non-invasive means to estimating patient-specific morphometric parameters in the deeper regions of the lung, which are critical for understanding gas exchange and optimizing pulmonary drug delivery. Each study contributes uniquely to understanding airway disease: imaging-based analyses of upper airway variability inform conditions like asthma and obstructive sleep apnea, while our distal lung modelling approach targets deeper pathologies such as emphysema and interstitial lung disease, where structural changes occur beyond the resolution of standard imaging.

The overall aim of this study is to predict the lung morphology and to identify the inter-subject lung morphology variability in healthy individuals. For this purpose, we present a gamma scintigraphy imaging study conducted on a cohort of six healthy individuals. For this purpose, a conventional dual head gamma camera (Siemens-Symbia E; Siemens Healthcare, Germany) with single-photon emission computed tomography (SPECT) facility at Sri Venkateswara Institute of Medical Sciences (SVIMS), Tirupati, India, was used. Radioaerosols were generated using commercially available Biodex Venti-scan® (Biodex Medical Systems, Inc., New York) radio-aerosol delivery system. This system is manufactured to be low-cost and for single-use, thereby minimizing the risk of cross-contamination and infection transmission between subjects. The retention of the radio-aerosol is used to calculate the regional lung deposition as well as the central to peripheral lung deposition ratio for the subjects under the study. By invoking a numerical model for aerosol transport, we estimated the distal lung morphometric parameters from the experimentally measured aerosol concentration.

Our analysis demonstrates that these morphometric parameters can be utilized to quantitatively determine airway length and diameter across all generations, estimate mean alveolar diameter, and derive critical metrics for personalized dosimetry assessment, including functional lung surface area and generation-specific deposition profile. This determines the bioavailability of the aerosol entering the human respiratory tract. This study opens a new era in personalized medicine by identifying patient-specific lung morphometric marker using a relatively non-intrusive method. The same study can be extended to patients with respiratory illness or in cases where targeted delivery of drugs is required. Once the morphological parameters are found for the infected individuals, tailoring the required dosage is easier.

## 2 Methods

### 2.1 Study design and participants

This study selected a cohort of six healthy individuals with no respiratory illness. Since the study focuses on identifying differences in lung morphometry (and not similarities) between the individuals, six is an adequate number of subjects for the cohort. Smokers are excluded from this study, as smoking can influence the characteristics of lung deposition. Study participants were chosen specifically to ensure they are free from respiratory symptoms like colds, sore throats, coughs. Healthy individuals who had pulmonary function tests within the normal range were selected. The study was conducted in accordance with relevant guidelines and regulations and was approved by the Institutional Ethics Committee (IEC) of Sri Venkateswara Institute of Medical Sciences (SVIMS), Tirupati, India, where the study was performed.

Written informed consent was obtained from all the participants prior to the study. A detailed history was taken in all the cases, and a thorough physical examination was carried out, especially focusing on the respiratory system. A chest radiograph (posterior-anterior view) was obtained for all subjects to ascertain healthy lung structure. Lung volumes were determined using the spirometry facility available at SVIMS, Tirupati, using the Morgan Transfer Test Benchmark Pulmonary Function Testing System (Morgan Scientific, Inc. Haverhill, MA, United States) as per the American Thoracic Society/European Respiratory Society guidelines (Miller et al., 2005; Bhakta et al., 2023). Anthropometric and spirometry data of the subjects under study are listed in Table 1.

**TABLE 1:** Anthropometric and spirometry data.

Subject	Age (yr)	Height (cm)	Weight (kg)	FEV1 (L)	FEV1% predicted	FEV1/FVC	FEV1/FVC % predicted
**1**	26	175	75	4.43	119	0.89	106
**2**	38	175	86	3.45	102	0.91	111
**3**	25	172	81	3.78	102	0.87	95
**4**	39	173	66	3.32	101	0.81	99
**5**	24	182	87	4.47	108	0.88	98
**6**	41	159	65	2.68	110	0.91	108

### 2.2 Aerosol administration and gamma camera image acquisition

In the gamma scintigraphy study, 99mTechnetium phytate radio-aerosol was generated using a Biodex Venti-scan® radio-aerosol delivery system. Tc-99m phytate is a low molecular substance which makes good aerosols for inhalation lung imaging similar to human serum albumin. Also m99-Tc-phytate is not absorbed from the pulmonary epithelium. Thus it is a suitable substance used for studying the mucociliary clearance function besides ventilation (Isawa, 1994). The mass median aerodynamic diameter (MMAD) of the aerosol produced by the nebulizer was about 0.5 μm. The radio-aerosol generated using the radio-aerosol delivery system was administered through a standard mouthpiece. The subject’s nose was clamped, and the subjects were instructed to breathe through their mouths.

The radio-aerosol solution used on all subjects contained 20 mCi of radioactivity. The subjects inhaled the aerosol for about 3 minutes so that about 2 mCi, i.e., one-tenth of total preparation, is deposited inside the thorax (Guleria et al., 2003). Subjects were also instructed to breathe slowly and deeply while inhaling radio-aerosol. Subjects were then made to lay in supine position comfortably on the examination table under the gamma camera. Radioactivity in the thorax was measured continuously in sequential frame mode for the first 2 hours. To obtain regional deposition from the radio-aerosol retention, the subjects were recalled again at two more-time instances after 4 h and 22 h to acquire the gamma-ray image. The procedure elaborated by Guleria et al. (2003) was followed in the image acquisition. Since the left lung is partially hidden by the heart and the stomach, the data corresponding to the right lung was used for all analysis and discussion. All the data collected were corrected for radioactive decay and background.

## 3 Results

### 3.1 Clearance of radio-aerosol, retention, and relative regional deposition

**FIGURE 1:**
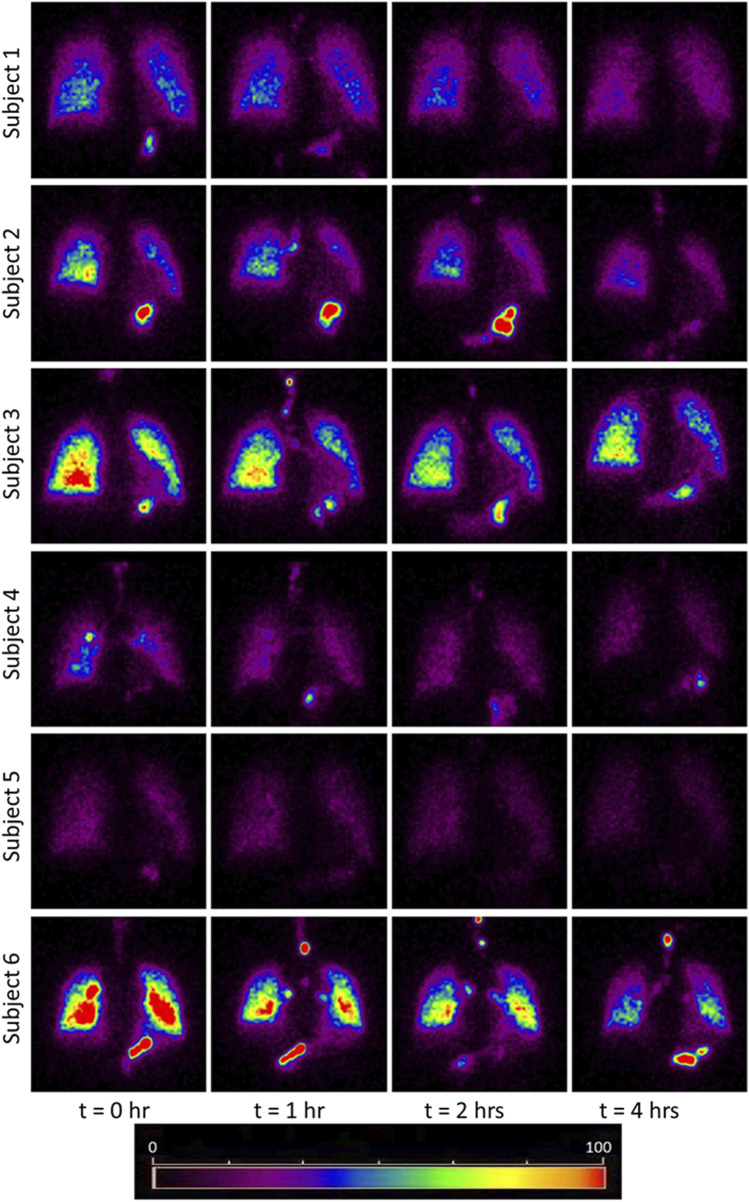
Images obtained using Gamma Camera at t = 0, 1, 2, 4 h. The red and yellow colored bright spots indicate that more aerosols have been retained in those regions. The bright spots in the stomach area due to the assimilation of the aerosol at the mouth walls. The trend of clearance kinetics can be observed from the reducing intensity at increasing time intervals. However, each image is unique for every subject and identifies the importance of inter-subject variability even among a small cohort healthy subject. Comparing the initial image of subject 5 with subject 3 or 6, it appears as though the subject 5 had inhaled about 1/4th of aerosol than the subject 3 and 6. Thus this study has attempted to bring out the morphometric cause of this variation. The considerable difference in brightness is a striking feature which emphasizes the need for personalized treatment.

The images obtained from the gamma camera for the subjects under the study have been shown in Figure 1. This figure shows the images taken by the camera at successive time instants. The first image acquired immediately after the subject inhaled the radio-aerosol is shown as the image at time, t = 0 h. Similarly, the images taken at t = 1h, t = 2h, and t = 4 h are also shown in the same figure. All the images in Figure 1 are pseudo-color coded to the same scale in order to effectively interpret the results and compare results across subjects. It must be mentioned that care was taken to ensure that each subject received the exact dosage of the radio-labeled aerosol, and the gamma camera gain setting was unchanged. We will begin by discussing the images from the six subjects obtained at t = 0 h. It can be observed that each subject has a qualitatively unique initial image, suggesting differing amounts of aerosol deposition. For example, the right lung in Subject 6 is nearly five times brighter than that of Subject 5. This variability is directly attributable to variation in extra-thoracic lung morphometry. During inhalation, some radio-aerosol gets into the digestive tract due to swallowing. Interestingly, this phenomenon manifests as bright spots visible in the gastrointestinal tract, particularly in the stomach (see Figure 1). The temporal variation in image patterns for each subject is analyzed over time. The clearance pattern of the radio-aerosol can be visualized from the reducing intensity in the images with time for all subjects. However, marked differences can be observed in the manner in which the aerosol clears out of the lung. These differences are discussed using quantitative measures.

Three quantities have been defined to quantitatively assess different characteristics of the deposition process: Retention of aerosol (Rt), Relative Regional Deposition (RRD), and Central to Peripheral deposition ratio (C/P).

**FIGURE 2:**
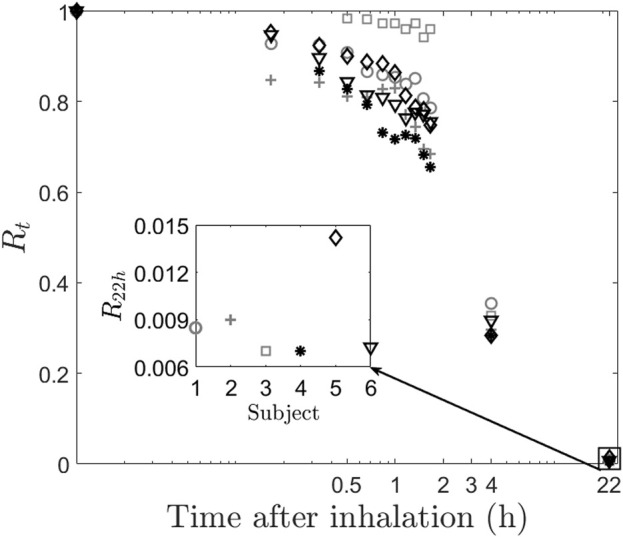
Clearance of radio-labeled particles from the lung post inhalation. The main figure shows the retention of aerosol for the subjects. The inset figure shows the retention after 22 h. Inter-subject variability can be clearly observed even at low values of retention.

**FIGURE 3:**
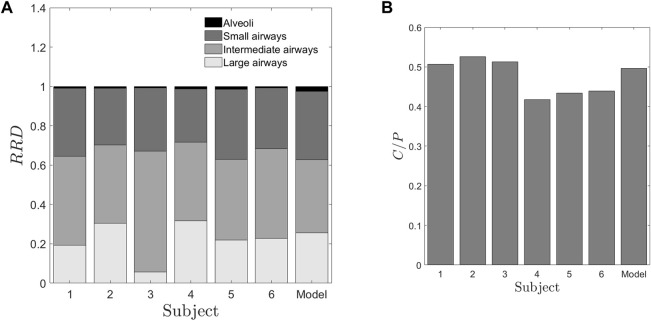
Deposition parameters extracted from the retention of radio-aerosol **(A)** Relative Regional Deposition (RRD) **(B)** Central to periphery C/P ratio. The abscissa numbers 1–6 represent the healthy subjects under the study. The abscissa “Model” represents the results from the numerical model^1^ with the lung dimensions given by Weibel and Weibel (1963) These two figures give us salient information that though the “Model” values, characterize more or less an average human, it fails to provide any data on inter-subject variability. Further these RRD values or the C/P ratios are not correlated to any of the spirometry data. In Figure 3A, the regions from the top of each bar black, dark grey, grey and light grey regions represent the relative deposition in alveoli, small, intermediate and large airways respectively.

Retention at time, t (Rt) forms the basis of calculation of the other two quantities. Rt is defined as the half-life corrected fraction of remaining radioactivity at time t. As expected, Rt shows a consistent decrease with time across all subjects, as depicted in Figure 2, which illustrates a plot of the normalized retention fraction against time. Remarkably, the clearance kinetics of the radio-labeled aerosol exhibits significant variability within the group. For example, Subject-3 retained the aerosol in the lung longer than Subject-4. In summary, the initial aerosol inhalation and its clearance characteristics differ within this smaller cohort of individuals. The clearance of the radio-labeled particles immediately after inhalation is slow (retention being high), and hence, there is low deposition in the upper respiratory regions. Thus, the initially cleared aerosol corresponds to the relative deposition in large airways. The clearance post 1.5 h of inhalation is, however, rapid (see Figure 2). This denotes aerosol deposition in the intermediate and the smaller airways. Retention after 22 h, $R_{22h}$ assumes that the bronchial ciliated airways are entirely cleared, and the remaining retention corresponds to the non-ciliated portion of the lung, which is alveolar deposition (Mortensen et al., 1994). We utilized these characteristics to define two quantities with the motive to classify individuals. Firstly, a Relative Regional Deposition (RRD) is calculated based on the retention (Rt) at different instants of time, as discussed by Sá et al. (2015) As the name indicates, this parameter indicates the relative deposition in different sections of the lung. The clearance of radiolabeled aerosols from the lungs at various time intervals is analyzed to determine the specific regions of aerosol deposition during inhalation. Figure 3a shows the Relative Regional Deposition (RRD) obtained from the gamma scintigraphy scan for the cohort. The last value on the abscissa named as ‘Model’ in the plot, is the value of RRD obtained using the numerical model of the human lung proposed by Karthiga et al. (2016) The mathematical model uses the lung morphometry of an ‘average’ individual as proposed by Weibel. The detailed calculation of the RRD and the C/P ratios from the numerical model is elucidated in the Supplementary Material.

### 3.2 Determination of C/P ratio using an intelligent threshold technique

In addition to RRD, the C/P ratio is calculated, representing the proportion of aerosol concentration deposited in the central (C) *versus* the peripheral (P) regions of the lung. The C/P ratio responds to changes in significant factors that influence the deposition characteristics in the lung, namely, particle size, inhaled airflow rate, and airway patency (Verbanck et al., 2020). The conventional clinical approach for determining the Central-to-Peripheral (C/P) ratio involves manually identifying a region of interest (ROI) on the right lung to distinguish between central and peripheral regions. Time-activity curves are then generated for each region. After applying corrections for decay and background, the C/P ratio of counts is calculated at each time point following the inhalation of the radio aerosol.

There are various methods available in the literature to draw these regions of interest (Biddiscombe et al., 2011; Alcoforado et al., 2018). One of the approaches to find the C/P ratio is where the central region comprises of one-third of the lung area, and the rest two-third region consists of the peripheral lung region (Smaldone et al., 1989). This method has been implemented in this study. To minimize user-induced variability in the process of ROI identification, this study employs an intelligent thresholding technique. This method segregates the lung into central and peripheral zones based on the area ratio of these regions.

### 3.3 Intelligent adaptive threshold technique for demarcating C/P boundary

The process of intelligent image threshold technique is completely automated in MATLAB®, thus reducing user-induced variability in the process of defining a region of interest (ROI). The first step in is to identify the lung boundary. For this purpose, the first image at the start of recording the data at t = 0 h is used. The grayscale level or the image threshold in MATLAB® has been automated for all the subjects based on pixel intensity to give the lung outline. For each subject, a threshold value which gives the maximum lung area is calculated. This particular threshold value can detect all the connected components of the image, giving rise to the maximum lung area.

**FIGURE 4:**
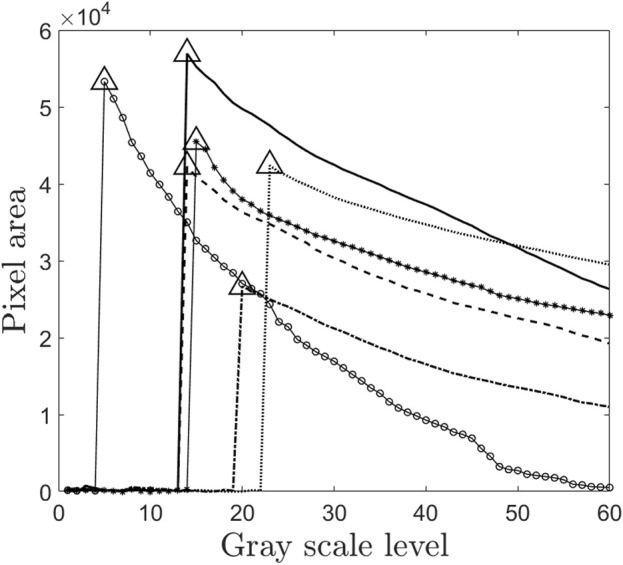
Plot of grayscale level and pixel value. This figure is the part of the intelligent threshold technique proposed for finding C/P ratio. The values with triangle markers are the optimal threshold levels which correspond to maximum lung area for each of the subjects. Thus this threshold value is used for finding the lung outline. Since this method is automated, it passes one of the most significant challenges in avoiding intra-subject variability in drawing ROI of gamma scintigraphy. Each marker in the figure represents the subjects 1 to 6 in the study.

Figure 4 presents a plot of the total number of pixels against the grayscale threshold levels for the study subjects. The pixel count, represented on the y-axis, corresponds to the right lung area. The triangle marker in the plot indicates the maximum right lung area and its associated grayscale threshold. The lung outline, which aligns with the maximum lung area, is extracted to define the lung region. Subsequently, the lung outline from the initial image is superimposed on the corresponding image acquired post-inhalation. Using image processing techniques in MATLAB®, the total lung area is then quantitatively determined.

**FIGURE 5:**
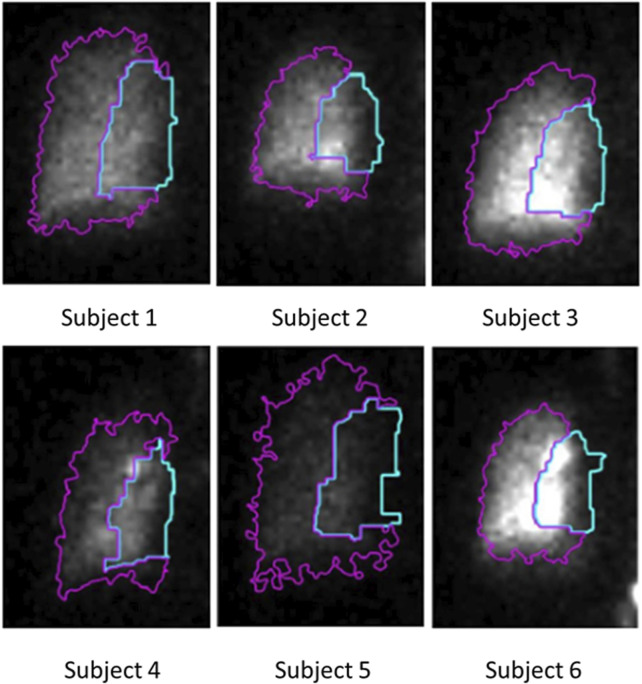
C/P outline obtained using the proposed intelligent threshold technique. The area enclosed by the blue line is the central zone of the lung. The area enclosed by the magenta-colored line is the peripheral zone of the lung. The ratio between the counts in these two zones is the C/P ratio.

This area is also divided into two distinct regions - the central region, comprising one-third of the area, and the peripheral region, comprising the remaining two-thirds (Biddiscombe et al., 2011). The C/P ratio derived from the initial image is depicted in Figure 3B, while Figure 5 illustrates the lung outline and the central-to-peripheral demarcations for the study subjects. On average C/P ratio across the subjects is approximately 0.5. Although this ratio identifies the approximate proportion of the aerosol reaching the peripheral zone, it does have a variability of about ±15% within the individuals under study. Further investigation is focused on identifying the morphological factors contributing to this variation.

### 3.4 Determination of subject-specific morphometric markers

The link between variability in lung morphology and aerosol deposition has not been experimentally studied in the literature and forms an essential motivator of the current work. For example, a study reported by Mauroy et al. (2004) concludes that branching structure of a bronchial tree plays a crucial role in determining the aerosol deposition characteristics. The authors also explain that minor structural changes among individuals could cause significant changes in respiratory system performance. Our previous study (Karthiga et al., 2016) also corroborates their findings and shows that small morphological changes of the lung from generation 5 terminating in the acinus, cause significant variability in alveolar deposition. Inter-subject variability has indeed been studied with the help of several computational models of the respiratory tract as well as aerosol dynamics, but no experimental data is available on healthy human subjects. We present the first study to characterize the variability in aerosol deposition in a cohort of healthy subjects. We present a simple approach to predict subject-specific distal lung morphometric parameters from the experimentally obtained aerosol deposition characteristics.

This study aims to identify subject-specific morphometric parameters that estimate the length and diameter of each generation. The length and diameter of the daughter airways at every generation for a “Weibel” individual used in the numerical model (Karthiga et al., 2016) were given by empirical equations by Weibel and Weibel (1963). The independent parameters in these empirical equations are listed in Table 1 of our previous study (Karthiga et al., 2016). $P_{1}$ and $P_{3}$ determine the length and diameter of the first 3 generations, respectively. Since our motivation is to find the dimensions of the lung closer to the alveolated end, the parameters $P_{1}$ and $P_{3}$ have been excluded. $P_{2}$ , $P_{4}$ and $P_{5}$ occur in exponential function in ; Karthiga et al., 2016). Due to the sensitive nature of these parameters, even minor changes resulted in exaggerated and unrealistic alterations to lung morphology. Therefore, these three parameters cannot vary significantly from individual to individual and are assumed to be at their Weibel values for all individuals. This narrows down our options to the two most critical parameters for personalization: $P_{8}$ and $P_{9}$ . These parameters determine the length and diameter of the airway, respectively, in generations 3 ≤ z ≤ 23.

These two parameters determine the airway volume without including the alveolar volume. To incorporate a quantitative descriptor of alveolar geometry, the alveolar diameter ( $d_{alv}$ ) is added as a parameter, augmenting our predictive acinar lung morphology model. Additionally, we have posed a constraint that the morphology determined by these parameters needs to be realistic. Finally, the parameter estimation procedure adds the Total Lung Capacity (TLC) as a subject-specific physiological constraint. TLC of the six individuals has been calculated using the prediction equations given by Vijayan et al. (1990). To identify the morphometric parameters, an error function, ε is postulated as a normed difference between the numerical predictions of ${\left(C/P\right)}_{m}$ ratio and experimentally determined values of C/P ratio. Specifically, $\varepsilon =\left|\frac{C}{P}-{\left(\frac{C}{P}\right)}_{m}\right|$ In this manner, the subject-specific lung morphometric parameters, $P_{8}$ , $P_{9}$ . and $d_{alv}$ have been estimated as those values where the error function is minimum. This minimization was performed using the optimization toolbox in MATLAB®. Error functions using RRD measurements were also deployed initially and the minimization process was repeated for each set of error functions. However, the parameters obtained by RRD methods were not robust and convergent.

**FIGURE 6:**
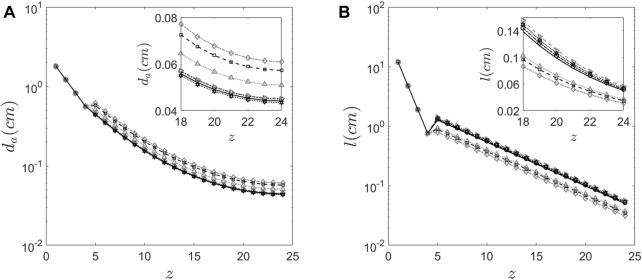
Morphology features of the human lung **(A)** Diameter of the airway predicted at every generation for the six subjects using the proposed method. **(B)** The length of the airway predicted at every generation for the six subjects using the proposed method.

The proposed method which uses C/P ratio is robust, convergent and predicts physically relevant values for the parameters. Although there are various factors or parameters that are influenced by inter-subject variability in morphometry, in this study we assume that only C/P ratio influences the lung morphology. The parameters obtained using the proposed method are tabulated in Table 2. The corresponding lengths and diameters of the bronchioles at various generations calculated using these parameters and Weibel’s morphometry model (Weibel and Weibel, 1963) are shown in Figures 6A,B. Table 2 shows the predicted parameters as well as their deviation from the Weibel values (in percentage terms).

**TABLE 2:** Parameters predicted applying the proposed method that uses C/P ratio.

Subject	Predicted parameters			% deviation from weibel value		
	$P_{8}$	$P_{9}$	$d_{alv}$	$P_{8}$	$P_{9}$	$d_{alv}$
1	2.59	1.33	0.0281	4	2	4
2	2.80	1.33	0.0289	12	2	6
3	2.69	1.28	0.0279	8	−2	2
4	1.56	1.79	0.0,286	−38	38	5
5	1.70	1.69	0.0290	−30	30	6
6	1.84	1.50	0.0269	−6	15	−1

Parameters predicted applying the proposed method that uses C/P ratio. The parameters obtained are physiologically relevant which is evident from the dimensions of lung plotted in Figure 6 using $P_{8}$ and $P_{9}$ . The values of $P_{8}$ and $P_{9}$ are used to calculate the length and diameter of every generation of the lung. With this information, the surface area of lung, alveolar volume and other morphological parameters related to drug uptake are calculated. This information pertaining to lung morphometry is not predicted by any other experimental or numerical or imaging modality.

**FIGURE 7:**
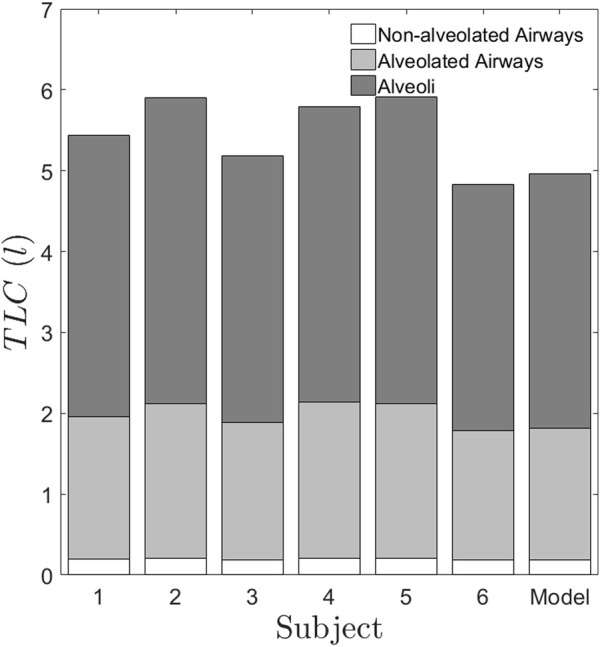
TLC predicted for the six subjects using the proposed method. The generations 0 < z < 17 represents the non-alveolated airways. The generations 17 < z < 23 represent the alveolated airways. The alveolated airways and alveoli contribute to differences in volume between subjects. It is to be noted that the non-alveolated region occupies a small volume in the lung, which is lesser than 0.5l. But the conventional imaging of the lung captures only this region and gives the lung dimensions which are greater than 2 mm, whereas our method predicts the smaller dimensions, which are sensitive to inter-subject variability.

Figure 6 graphically depicts the variation of bronchiole diameters and lengths at each generation in the six subjects. The average diameters and lengths of a model individual are also plotted in the same figure for reference. The numerical approach predicts that subjects 4 and 5 have larger diameter bronchioles but are shorter in length. In contrast, subjects 2 and 3 have smaller diameter bronchioles but are longer. One could corroborate this information with the corresponding images in Figure 1 to see that subjects 2 and 3 have higher aerosol deposition, while subjects 4 and 5 have a lower aerosol deposition. The increased wall surface area due to longer bronchioles appears to be an internally self-consistent explanation of this observation. The inset in Figures 6A,B show the lengths and diameters of the alveolated airways only. Even though the parameters $P_{8}$ and $P_{9}$ vary about 30% from the Weibel values for subjects 4 and 5 (see Table 2), the lengths and diameters are physically consistent for all the six subjects (see Figure 6). Total Lung Capacity (TLC) is another physiologically relevant parameter. Table 3 also presents the deviation of other functional aspects of the lung. It can be observed that the non-alveolated airway volume (0 < z < 17) almost remains same for all subjects, while the alveolated airway volume (17 < z < 23) shows significant subject-to-subject variability in contributing to the total lung capacity (see Figure 7 which shows TLC for all the subjects). Therefore, variation in alveolar volume is a prime cause of inter-subject variability in TLC, and hence aerosol deposition. The parameter $d_{alv}$ (which is a measure of the mean alveolar diameter) shows only about 1%–6% variation for all subjects under the study (see Table 2). The corresponding alveolar volume varies by up to 21% (see Table 2). Thus, aerosol deposition is sensitive to alveolar volume. Furthermore, our approach successfully predicts morphometric parameters for distal airway generations (17 < z < 23), which are currently inaccessible through conventional imaging techniques.”

**TABLE 3:** Change in surface area and lung volumes in relation to Weibel values.

Subject	% change in predicted surface area	% change in predicted airway volume	% change in predicted alveolar volume
1	6	8	11
2	−3	17	20
3	4	4	5
4	6	18	16
5	−8	17	21
6	−6	−2	−3

The deviation of lung surface areas and total lung volumes for each subject from an average individual is a simple visual way of presenting inter-subject variability data (see Table 3). Lung surface area is an important factor influencing drug deposition. For example, the change in surface area for subject 5 amounts to about 8%. Since the surface area for subject 5 is reduced by 8%, the drug absorption would also be reduced in case of subject 5 than the average individual. Surprisingly, the percentage change in alveolar volumes for each subject is considerably higher than the volume changes of the airway passages. We, therefore, show that alveolar volume, while only being about one-third of the total lung capacity, is a primary cause of inter-subject variation in morphometry. As the lung surface area and alveolar volume are fundamentally important parameters in drug delivery and absorption, it is essential to estimate these parameters for an individual subject. Therefore, predicting the parameters would cater to the needs of both the patient and the physician in personalizing dosage and therapy.

## 4 Discussion

*In vivo* radiographic imaging techniques are proving to be efficient methods for estimating drug deposition *via* many routes of administration (Mairinger et al., 2022). 3D-radiographic imaging techniques include SPECT, positron emission tomography (PET) and MRI while two-dimensional radiographic imaging techniques include gamma scintigraphy. Though 3D technologies offer certain advantages over gamma scintigraphy, they are primarily outweighed by the radiation dosage requirement (Newman and Wilding, 1999; Xu et al., 2024). From this perspective, gamma scintigraphy continues to be the standard protocol for measuring aerosol deposition (Kwok et al., 2019) due to its simplicity and lower radiation dose (Conway, 2012). In the current study, we have used this technique to measure regional lung deposition as well as C/P ratio. To the best of our knowledge, this is the first study wherein an imaging modality with minimal radiation exposure is being used to estimate morphometric parameters of the lung structure. The procedure followed herein has the potential to evolve into a walk-in lab test, where a subject undergoing a conventional gamma scintigraphy scan or ventilation study, can additionally obtain his/her lung morphology map from the predicted parameters.

This study is also relevant for advancing the science on inter-subject variability of aerosol dynamics. The overall trend of aerosol clearance in all the subjects remains qualitatively the same as can be observed in Figure 2. However, each individual has certain distinct features that make the retention values different, especially during the first 2 hours. We show that the morphology variability is a critical underlying cause of these changes in retention. One more important aspect to be noted from Figure 2, is the inset figure showing the retention at 22 h. As expected, the retention is very low since the aerosol size, which we have used is 0.5mm, which falls in the intermediate particle size, giving rise to low alveolar deposition (Karthiga et al., 2016). However, it is surprising to note that even at such low values of retention $R_{22h}$ we are clearly able to see the difference between subjects. For example, subject 5 shows higher retention of aerosol at 22 h, whereas subject 3 and 4 show least $R_{22h}$ with nearly 100% difference between them. Finally, we show that the alveolar deposition value does not show correlation with other confounding factors such as height, weight, and TLC of the subject.

From Figure 3A, the relative depositions in all the subjects for all the regions, i.e., large, intermediate, small airways and alveoli differ significantly from one another. Similar to retention, these factors do not show correlation with any of the confounding factors such as height, weight or TLC of the subjects. The central to peripheral, C/P ratio obtained from the initial image is shown in Figure 3B. From this figure, it can be clearly seen that the value of C/P differs from individual-to-individual giving rise to inter-subject variability between healthy individuals. Meanwhile one more important aspect is to be noted from Figure 3. The model (Weibel) individual’s RRD and C/P ratio are like the average RRD and C/P ratio of the subjects under the study. Thus, Weibel morphometry data can be construed to represent an average individual without including the variability of morphology between individuals. Though Weibel data has been used as being representative of the entire population, it still does not help in personalized medicine. With the aid of the predicted parameters (see Table 2), other clinically relevant parameters such as regional deposition, total lung volume, total alveolar volume as well as functional surface area can be calculated (see Figures 3, 7 as well as Table 3). Thus, clinically relevant subject-specific quantities linked to bioavailability of an inhaled drug can be predicted.

The alveolated region of the lung is the region, which is rich in supply of blood vessels with the blood-gas barrier being at an average thickness of 0.3 μm (West, 2012). The alveolar deposition is therefore directly linked to systemic bioavailability. The increased acinar surface area is one of the critical factors influencing the drug bioavailability after deposition. From the results in Table 3, the acinar surface area of the subjects 1 and 4 is higher by 6% when compared to the average individual. Hence, drug absorption is likely to be higher for this subject than other subjects. Similarly, the dose delivered for subject 5 is lower than that for the average individual since the surface area is reduced by about 8%. In addition, the generation wise deposition dosage could significantly differ from that for an average individual (refer Table 2 for the values of $P_{8}$ and $P_{9}$ ) for each of the subjects. Similarly, for the subject 1, the change in lengths and diameters resulting from $P_{8}$ and $P_{9}$ is small in comparison to the average individual as seen in Table 2 and Figures 6A,B. But these variations contribute to only about 6% change in surface area. Hence, the bioavailability of any drug delivered to subject 1 might not be same as that of the average individual, even though the generational deposition might be the same. This crucial insight, attainable only through the synergistic combination of gamma camera imaging and our mathematical modeling, holds significant clinical implications and underscores the value of this interdisciplinary approach.

### 4.1 Strengths and limitations of the study

The prime objective of this study is to address the problem of inter-subject variability and is it possible to address it practically. This study has considerably addressed this issue and has developed a method of estimating the inter-subject variability in deposition. The study has several assumptions that can be considered as strengths.

1. This study attributes inter-subject variability to dimensional and morphometry changes of the respiratory tract.
2. Other factors such as biological and immunological factors of the individuals have not been considered.
3. The study also uses a simple yet powerful one-dimensional model of human lung that includes all complex phenomenon of transport and deposition of aerosols (might be both drug as well as microorganisms).

Usage of such idealizations here are more important because complex simulations of various other factors require lot of computational power that is not required at this context.

### 4.2 Sample size considerations

An important disclaimer at the outset of this study is that, this is not an epidemiological study where in sample size need to be calculated at the planning stage. This is a preliminary study that has attempted to bridge deposition variability directly to morphological variability. No statistical analysis was performed on this pilot study due to the limited sample size of six participants thus precludes statistical inference. The results can be interpreted as proof-of-concept data that demonstrate the technical feasibility of the approach in estimating inter-subject variability. Also one needs to understand the ethical considerations on performing such a study involving radiation exposure on healthy individuals. Hence the sample size is fixed to be small. If the proof of method is established then only the usage of available scanned images from patients with lung diseases can be used with the same method to predict airway morphometry. It fits within the scope of pre-clinical research and provide a base for using the same on larger set of cohorts. It is also relevant to the current research topic on human airways classification for diseases.

## 5 Conclusion

In this study, we have built upon conventional gamma scintigraphy (as detailed in the methods and results section) of taking images at t = 0 h, t = 4 h, and t = 22 h to calculate both RRD and the C/P ratio. However, we have found that the method using C/P ratio predicts physiologically consistent values of the parameters as well as passes the convergence and robustness tests. The study has predicted the morphometry map of the subjects under the study. Since the C/P ratio method using an intelligent threshold technique method only relies on the initial image of gamma scintigraphy images, the overall test could be further shortened, and the radiation dosage decreased even more. It is also possible that the radio-isotopes could have shorter half-lives sufficient enough to get these two images rather than to record 24-h retention. This further reinforces the fact that research in this direction is likely to bear clinically relevant results affecting bedside outcomes through both intelligent drug design as well as dosage control, personalized for a given individual.

Although there are various factors or parameters that are influenced by inter-subject variability in morphometry, in this study we assume that only C/P ratio influences the lung morphology. Previous ADAM or airspace dimension studies (Gebhart et al., 1981; Brand et al., 1995; Brand et al., 1999; Blanchard, 1996) have solely utilized mouth-exhaled and inhaled aerosol bolus measurements, whereas approach used in this study employs gamma scintigraphy-based measurements. Consequently, airway dimension estimates derived from this protocol are likely to offer enhanced reliability and robustness. It may be most useful to classify individuals as “dilated,” “constricted,” *etc.* The classification problem is likely to be more robust than a parameter estimation problem.

The methodology presented in this study holds potential for translation into a walk-in laboratory test, wherein a subject undergoing a standard imaging procedure could simultaneously obtain a quantitative map of lung morphometry based on the predicted parameters. Furthermore, the required instrumentation is already available and accessible in standard clinical settings, making the approach feasible for routine implementation in outpatient diagnostics.

## Data Availability

The raw data supporting the conclusions of this article will be made available by the authors, without undue reservation.
