# Supplementary material for: Patient-specific estimation of distal lung morphometry using aerosol deposition data
Source: Front Physiol. 2025 Jul 29;16:1611545. doi: 10.3389/fphys.2025.1611545 (PMC12339505; doi:10.3389/fphys.2025.1611545)
Supplement: Supplementary file 1 [file DataSheet1.pdf]

Supplementary Material

Patient-specific estimation of distal lung morphometry using aerosol deposition data

Karthiga Devi, S.G [1,2*], Mohan Alladi [3], Kalawat T.C [4], Mahesh V Panchagnula[1]

[1]Department of Applied Mechanics and Biomedical Engineering, Indian Institute of Technology Madras, Chennai

[2]Department of Environmental Health Engineering, Sri Ramachandra Institute of Higher Education and Research, Chennai.

[3]Department of Medicine, Sri Venkateswara Institute of Medical Sciences, Tirupati

[4]Department of Nuclear Medicine, Sri Venkateswara Institute of Medical Sciences, Tirupati

*** Correspondence:**
Corresponding Author
karthigadevi25@gmail.com

1 Supplementary Data

1.1 Retention and Relative Regional Deposition (RRD)

Relative Regional Deposition (RRD) is calculated based on R_t the retention R_t at different time points. Retention at time t, R_t is defined as the half-life corrected fraction of remaining radioactivity at time t. The retention R_t at any given time t can be used for quantifying the regional deposition along different generations of the airway tree (1). The following terms have been defined and calculated based on the retention, R_t

- Relative deposition in the large size airways, $L = 1 - R_{1h30min}$
- Relative deposition in the intermediate size airways, $I = R_{1h30min} - R_{4h}$
- Relative deposition in smallest bronchial airways, $S = R_{4h} - R_{22h}$
- Relative deposition in alveoli, $A = R_{22h}$

The Relative Regional Deposition (RRD) quantities (LISA) obtained from the gamma scintigraphy scan for the six individuals are shown in figure 3(a) of the manuscript.

1.2 Calculation of Regional deposition and C/P ratio calculation from numerical model

In case of the numerical model, the same relative deposition in the large (L_m), intermediate (I_m), small (S_m) airways and alveoli (A_m) has been calculated from using the numerical model (2) elucidated

below. The large airways are defined to be from the first bronchus as it enters the left and right lung to the 4th generation. Thus, the relative deposition in the large airways (L_m), is given by,

$$L_m = \frac{\frac{1}{5T}\int_{9T}^{13T}\int_{1}^{4} l(z)L_p(z,t)\,dz\,dt}{\frac{1}{5T}\int_{9T}^{13T}\int_{1}^{23} l(z)L_p(z,t)\,dz\,dt} \qquad (1)$$

Similarly, we have defined the intermediate airways to be from the 5th generation to 17th generation. Thus, the relative deposition in the intermediate airways (I_m) is given by,

$$I_m = \frac{\frac{1}{5T}\int_{9T}^{13T}\int_{5}^{17} l(z)L_p(z,t)\,dz\,dt}{\frac{1}{5T}\int_{9T}^{13T}\int_{1}^{23} l(z)L_p(z,t)\,dz\,dt} \qquad (2)$$

The small airways have been defined to be from the 18th generation to 20th generation. Thus, the relative deposition in the small airways (S_m) is given by,,

$$S_m = \frac{\frac{1}{5T}\int_{9T}^{13T}\int_{18}^{20} l(z)L_p(z,t)\,dz\,dt}{\frac{1}{5T}\int_{9T}^{13T}\int_{1}^{23} l(z)L_p(z,t)\,dz\,dt} \qquad (3)$$

The relative deposition in the alveoli has been defined to be engulfed in the alveolar sacs of 21st to 23rd generations. Thus the relative deposition in the alveoli (A_m) is given by

$$A_m = \frac{\frac{1}{5T}\int_{9T}^{13T}\int_{21}^{23} l(z)L_p(z,t)\,dz\,dt}{\frac{1}{5T}\int_{9T}^{13T}\int_{1}^{23} l(z)L_p(z,t)\,dz\,dt} \qquad (4)$$

Apart from regional deposition, the central to peripheral region deposition ratio obtained from the scintigraphy scan can be compared with that obtained from numerical model. This is possible by defining the central and peripheral regions for the model. Gamma scintigraphy is a planar imaging method where a 3-D organ is imaged via 2-D method. Since the imaging modality used herein is planar, the central region overlaps some of the peripheral region and vice versa. The radioactivity counts from some of the peripheral region would add up to give the deposition in the central region. To account all these effects, we have defined central region from the first bronchus as it enters the left and right lung to the 15th generation. The peripheral region is from the 15th generation to the end of 23 generations. The $\frac{C}{P}$ratio in the model is calculated as follows,

$$\frac{C}{P_m} = \frac{\frac{1}{5T}\int_{9T}^{13T}\int_{3}^{15} l(z)L_p(z,t)\,dz\,dt}{\frac{1}{5T}\int_{9T}^{13T}\int_{16}^{23} l(z)L_p(z,t)\,dz\,dt} \qquad (5)$$

Thus, using the above procedure, the RRD parameters L_m, I_m, S_m airways and alveoli A_m airways and $\frac{C}{P_m}$ from the model has been calculated.

2 References

1. Sá RC, Zeman KL, Bennett WD, Prisk GK, Darquenne C. Effect of Posture on Regional Deposition of Coarse Particles in the Healthy Human Lung. J Aerosol Med Pulm Drug Deliv. 2015 Dec;28(6):423–31.

2. Karthiga Devi SG, Panchagnula MV, Alladi M. Designing aerosol size distribution to minimize inter-subject variability of alveolar deposition. Journal of Aerosol Science. 2016 Nov 1;101:144–55.
